# Preparation and Photocatalytic Performance of Sodium Alginate/Polyacrylamide/Polypyrrole-TiO_2_ Nanocomposite Hydrogels

**DOI:** 10.3390/polym15204174

**Published:** 2023-10-20

**Authors:** Amatjan Sawut, Tongmeng Wu, Rena Simayi, Xueying Jiao, Yurou Feng

**Affiliations:** State Key Laboratory of Chemistry and Utilization of Carbon Based Energy Resources, College of Chemistry, Xinjiang University, Urumqi 830017, China; wutongmeng0509@163.com (T.W.); lanmanxx98@163.com (X.J.); 13546312689@163.com (Y.F.)

**Keywords:** sodium alginate, nanocomposite hydrogel, titanium dioxide, photocatalysis, dye wastewater treatment

## Abstract

The application of photocatalysis technology in environmental pollution treatment has garnered increasing attention, and enhancing the photocatalytic efficiency and recyclability of photocatalysts represents a pivotal research focus for future endeavors. In this paper, polypyrrole titanium dioxide nanocomposite (PPy-TiO_2_) was prepared using in situ polymerization method and dispersed in sodium alginate/polyacrylamide (SA/PAM) hydrogel matrix to prepare SA/PAM/PPy-TiO_2_ nanocomposite hydrogels. The nanocomposite hydrogels were characterized by XPS, FT-IR, XRD, TGA, SEM, and TEM. The results showed that the composite materials were successfully prepared and PPy-TiO_2_ was uniformly dispersed in the hydrogel matrix. The incorporation of PPy in the SA/PAM/TiO_2_ composite hydrogel resulted in enhanced visible light absorption, reduced recombination efficiency of photoelectron-hole pairs in TiO_2_, and facilitated the photocatalytic degradation of methylene blue (MB) and methyl orange (MO) under sunlight irradiation. The photocatalytic efficiency of the composite hydrogel for MB was nearly 100%, whereas for MO, it reached 91.85% after exposure to sunlight for 120 min. In comparison with nano-TiO_2_ and PPy-TiO_2_, the SA/PAM/PPy-TiO_2_ nanocomposite hydrogel exhibited a higher degradation rate of MB and demonstrated ease in separation and recovery from the reaction solution. Furthermore, even after undergoing five cycles of recycling, there was no significant decrease observed in photodegradation efficiency.

## 1. Introduction

The source of life and the link that connects all living beings on this planet is water. However, with the rapid progression of industries and population growth, water pollution has emerged as an urgent concern [1,2]. Consequently, the control and remediation of water pollution have become imperative issues for human society, necessitating immediate resolution [3,4]. Among various wastewater treatment technologies [5,6], the implementation of multiphase photocatalytic reactions utilizing semiconductor oxides as photocatalysts exhibits distinctive characteristics, including operation at ambient temperature, deep mineralization, and utilization of sunlight as an activating light source to directly drive the redox reaction. These features make it an ideal technology for wastewater treatment [7,8]. Titanium dioxide (TiO_2_) is one of the most extensively studied semiconductor photocatalysts due to its stability, high activity, low cost, and non-toxicity, showing great potential in remediating environmental pollution [9]. However, TiO_2_ can only excite electrons under ultraviolet irradiation (which accounts for 4% of total solar energy) due to its relatively wide band gap, greatly limiting its application in photocatalysis [10]. To address these limitations, researchers have employed various methods to modify TiO_2_, such as metal/metal oxide loading [11], metal doping [12], non-metal doping [13], dye sensitization [14], and coupling with other semiconductors [15]. Although these strategies can improve the optical response range or increase quantum yield to some extent, they are insufficient in enhancing photocatalytic activity, particularly in the visible light region.

In recent years, the utilization of conductive polymer materials for TiO_2_ modification has attracted increasing attention [16,17]. Among these materials, polypyrrole (PPy) stands out due to its ease of preparation, cost-effectiveness, and strong visible light absorption capacity [18,19,20]. PPy can serve as a stable photosensitizer to modify TiO_2_, broadening its spectral absorption range and enhancing its photocatalytic activity under visible light irradiation [21]. This is attributed to the reduction in the band gap of TiO_2_ by PPy, resulting in a smaller band gap energy value for PPy-TiO_2_ nanocomposites compared to pure TiO_2_ [22]. Moreover, the combination of PPy-TiO_2_ with hydrogel exhibits superior efficacy in addressing environmental issues associated with dye wastewater treatment. Li et al. [23] reported the fabrication of Macroporous polypyrrole (PPy)-TiO_2_ composites via in situ oxidative polymerization of pyrrole in the macropores of TiO_2_. The incorporation of polypyrrole enhances visible light absorption and facilitates the efficient separation of photogenerated electrons and holes.

Moreover, when the catalytic material is scaled down to the nanometer level, a significant enhancement in catalytic efficiency can be achieved [24]. Nano TiO_2_ possesses several advantageous properties such as small particle size, large specific surface area, strong magnetism, excellent photocatalysis and adsorption performance, good thermal conductivity, and dispersion characteristics [25]. These attributes have led to its extensive utilization in various fields, including environmental protection, energy production, information materials development, medical care, and other aspects [26]. However, the reduced size also poses challenges for the reusability of nano TiO_2_ particles in aqueous solutions due to difficulties associated with their separation and recovery processes, resulting in low material reuse rates [27]. Therefore, it becomes imperative to synthesize supported modified TiO_2_ nanoparticle photocatalysts.

Hydrogel is a type of gel with water as the dispersion medium, which consists of a hydrophilic homopolymer or copolymer crosslinked via physical or chemical means to form a three-dimensional network structure [28]. The utilization of hydrogels as adsorbents for the treatment of heavy metals and organic pollutants in wastewater exhibits distinctive characteristics, including simplicity in operation, facile modification, high adsorption capacity, and reusability [29]. Moreover, hydrogels possess translucency, porosity, and softness while enabling continuous photochemical transformations within an aqueous environment [30]. Consequently, hydrogels have emerged as promising carriers for nanoparticles in recent years. By effectively integrating hydrogels with photocatalysts, composite hydrogels with adsorption–photocatalytic functions have been achieved [30,31,32]. The presence of a hydrogel carrier facilitates the maintenance of structural integrity for photocatalysts during repeated cycles, thereby allowing easy removal of the composite photocatalyst from the reaction solution to prevent secondary contamination [33]. Furthermore, the flexibility endowed by the hydrogel imparts excellent mechanical properties to the composite material that enable folding and bending actions suitable for deployment in complex environments and confined spaces.

In this study, the unique properties of nano TiO_2_, PPy, and hydrogel were synergistically combined while drawing upon previous research methodologies and achievements [34,35]. A class of SA/PAM/PPy-TiO_2_ nanocomposite hydrogels that exhibit visible light responsiveness, high photocatalytic efficiency, excellent adsorption performance, and facile recyclability was successfully designed and synthesized. The potential application of these nanocomposite hydrogels in the adsorption–photocatalytic removal of harmful dyes from wastewater was investigated. The photocatalytic activity of these composite hydrogels was systematically studied under varying PPy content and pH conditions. Furthermore, the SA/PAM/PPy-TiO_2_ nanocomposite hydrogels exhibited enhanced photocatalytic efficiencies compared to nanoparticle TiO_2_ and PPy-TiO_2_ under sunlight irradiation. Additionally, the cyclic performance and mechanical properties of the composite hydrogel were evaluated to assess its practical applicability in real environmental scenarios. This design and synthetic strategy for adsorption–photocatalytic functionalized composite hydrogels provides a novel approach towards the construction of highly efficient, stable, visible light-responsive photocatalytic materials for treating organic pollutants.

## 2. Materials and Methods

### 2.1. Materials

Materials: Sodium alginate (SA, average molecular weight Mw: 2.8 × 10^5^ g/mol) was purchased from Tianjin Guangfu Fine Chemical Research Institute (Tianjin, China); acrylamide (AM) was purchased from Chengdu Kelong Chemical Reagent Factory (Chengdu, China); pyrrole (Py) and titanium dioxide (TiO_2_, anatase, with an average particle size of 25 nm; the band gap was about 3.2 eV) were purchased from Shanghai Maclean Biochemical Technology Co. (Shanghai, China); N,N-methylenebisacrylamide (MBA), ferric chloride and calcium chloride were purchased from Tianjin Zhiyuan Chemical Reagent Co., Ltd. (Tianjin, China); methyl orange (MO) and basic magenta (BM) were purchased from Tianjin Yongsheng Fine Chemical Co. (Tianjin, China); methylene blue (MB) and malachite green (MG) were purchased from Tianjin Xinbote Chemical Co. (Tianjin, China).

### 2.2. Preparation of Polypyrrole-TiO_2_ Nanocomposite

The polypyrrole-TiO_2_ (PPy-TiO_2_) nanocomposite was synthesized via in situ polymerization of pyrrole monomer and TiO_2_ nanoparticles in the presence of ferric chloride (FeCl_3_) as an oxidizing agent [36]. First, TiO_2_ was added to 1 M of HCl aqueous solution and placed into an ice water bath after ultrasound for 1 h. Then, a certain amount of pyrrole monomer was added and stirred continuously for 2 h to make the pyrrole monomer fully adsorbed on the surface of TiO_2_. In addition, a certain amount of FeCl_3_ was fully dissolved in 1 M HCl aqueous solution, where the molar ratio of pyrrole monomer and FeCl_3_ was maintained at a 1:2 ratio. The FeCl_3_ acid solution was slowly dropped into the mixed acid solution of TiO_2_ and pyrrole monomer, and after continuous agitation for 4.5 h, filtration, washing, and drying, PPy-TiO_2_ material was obtained. According to the above method, the PPy-TiO_2_ composites were prepared using different additive amounts of pyrrole, and the molar ratios of PPy:TiO_2_ were 0; 1:125; 1:100; 1:75; 1:50, respectively.

### 2.3. Preparation of Sodium Alginate/Polyacrylamide/Polypyrrole-TiO_2_ Nanocomposite Hydrogel

The specific production steps of the SA/PAM/PPy-TiO_2_ nanocomposite hydrogel are as follows: 0.1 g PPy-TiO_2_ is added to distilled water and ultrasonicated for 30 min to obtain a uniformly dispersed solution. After that, 0.2 g SA powder and 1 g AM were added into the evenly dispersed PPy-TiO_2_ solution, followed by 0.001 g crosslinker MBA, and stirred evenly. After that, an ultrasound was performed to make TiO_2_ evenly dispersed in the mixed solution. Then, the obtained solution was transferred to the glass mold, sealed, and placed under sunlight to promote the polymerization of AM. After the reaction was completed, the nano-TiO_2_ that was not firmly bonded on the surface was cleaned and immersed in calcium chloride (2 wt%) solution for 12 h to chelate calcium ions with sodium alginate. After that, the SA/PAM/PPy-TiO_2_ nanocomposite hydrogel was rinsed with a large amount of deionized water (in order to maintain the uniformity of sunlight intensity, we conducted the experiment at the same time every sunny day (August to September 2022, March to June 2023, 13 to 16 o’clock every day, Urumqi, China). In addition, to account for daily differences in light intensity, we conducted three parallel experiments during the experiment, each time at the same time of day).

### 2.4. Characterization

Before analysis, the prepared SA/PAM/PPy-TiO_2_ nanocomposite hydrogel and other samples were sufficiently freeze-dried and ground into powder. The adequately dried SA, PPy-TiO_2_, and SA/PAM/PPy-TiO_2_ hydrogel powders were analyzed using Fourier transform infrared spectroscopy (FTIR) on a Fourier transform infrared spectrometer (VERTEX 70 RAMI, Bruker, Bremen, Germany) from 4000 to 500 cm^−1^ using the potassium bromide compression method. Thermogravimetric analysis of the SA/PAM/PPy-TiO_2_ hydrogel powders was performed using a thermogravimetric analyzer (TG) (STA 7300, Hitachi, Tokyo, Japan) in nitrogen at a temperature range of 30 °C to 600 °C. The nitrogen flow rate was 200 mL/min, and the heating rate was 10 °C/min; TiO_2_, PPy-TiO_2,_ and SA/PAM/PPy-TiO_2_ hydrogel were then characterized using an X-ray powder diffractometer (XRD) (D8 Advance, Bruker AXS GmbH, Karlsruhe, Germany). The X-ray photoelectron spectrometer (XPS) analysis of TiO_2_, PPy-TiO_2,_ and SA/PAM/PPy-TiO_2_ hydrogel was carried out using ESCALAB 250Xi, Thermo Fisher Ltd., Waltham, MA, USA. After gold spraying the SA/PAM/PPy-TiO_2_ hydrogel, the morphology was analyzed using a field emission scanning electron microscope (SEM) (SU 8010, Hitachi, Japan) at various magnifications, and elemental analysis was performed using its own energy spectrometer (EDS). The microscopic morphology was observed using a high-power transmission electron microscope (TEM) (JEM-2100, Japan Electronics, Tokyo, Japan).

### 2.5. Swelling Performance Test of Composite Hydrogels

The swelling rate (SR) of SA/PAM/PPy-TiO_2_ composite hydrogel was measured by calculating the weight change of the hydrogel before and after full swelling. The prepared SA/PAM/TiO_2_ composite hydrogel was cut into cubes with length, width, and height of 10 mm, 5 mm, and 1 mm, respectively, and dried for use. At room temperature, the composite hydrogel was placed in a beaker containing 300 mL of deionized water and weighed after soaking. The swelling rate SR (g/g) of the SA/PAM/TiO_2_ composite hydrogel was calculated using the following equation:

(1) $$SR(g/g)=\left(W_{t}-W_{0}\right)/W_{0}$$

where W_0_ and W_t_ are the mass of the composite hydrogel before water absorption and the mass of the composite hydrogel at water absorption t.

### 2.6. Mechanical Property Test of Composite Hydrogels

SA/PAM/PPy-TiO_2_ composite hydrogel was cut into a dumbbell shape of 40 × 10 × 1 mm and tested using an electronic universal tensile testing machine with a tensile rate of 50 mm/min. The tensile stress and strain were calculated based on the initial cross-sectional area and initial length of the tested sample.

### 2.7. Photocatalytic Performance Test of Composite Hydrogels

SA/PAM/PPy-TiO_2_ nanocomposite hydrogel solar photocatalysis experiments were also divided into dark adsorption reactions and photocatalytic degradation reactions. First, the composite hydrogel was mixed with the dye solution and placed on the agitator to stir evenly until the adsorption equilibrium was reached, and then the mixture was exposed to sunlight together with the stirring device to start the photodegradation reaction. The specific steps are as follows:

Before the photocatalytic reaction, the SA/PAM/TiO_2_ composite hydrogel was cut into cubes with length, width, and height of 10 mm, 5 mm, and 1 mm, respectively, and dried for use. The photocatalytic experiments were divided into dark adsorption reactions and photocatalytic degradation reactions. The dark adsorption reaction was used to put 10 mg of the composite material into a quartz reaction tube containing 5 mL of 10 mg/L dye solution, and then the quartz reaction tube was placed on a magnetic stirrer to stir evenly so that the composite hydrogel and the dye solution were fully in contact until the adsorption equilibrium was reached. After the photocatalytic degradation reaction, the quartz reaction tube was placed under the 35 W LED lamp to radiate. After the photocatalytic experiment, the absorbance of the dye solution was measured using an ultraviolet spectrophotometer at regular intervals. The removal rate R (%) of dye in dye solution was calculated according to the following formula:

(2) $$R=\left(c_{0}-c_{t}\right)/c_{0}\times 100\%$$

where c_0_ and c_t_ are the initial dye concentration and the dye solution concentration A_t_ at time t, and A_0_ and at are the initial dye solution absorbance and the dye solution absorbance at time t.

## 3. Results and Discussion

### 3.1. Structural Characterization Analysis of Materials

**Figure 1:**
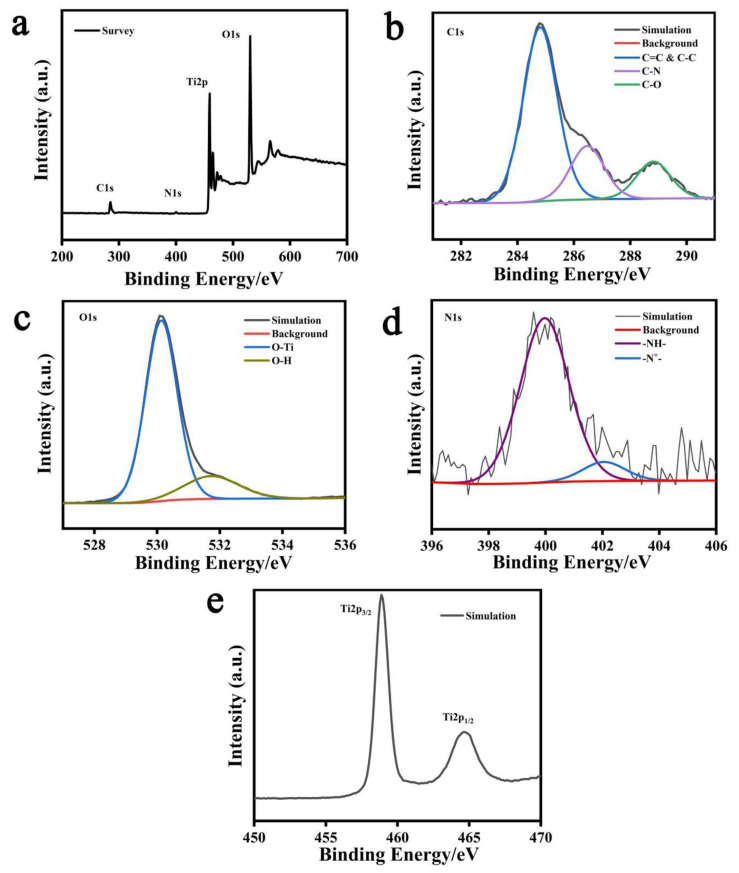
(**a**) XPS total spectrum, (**b**) C1s spectrum, (**c**) O1s spectrum, (**d**) N1s spectrum, and (**e**) Ti2p spectrum of PPy-TiO_2_.

X-ray photoelectron spectroscopy (XPS) was used to characterize the surface chemical composition of the PPy-TiO_2_ nanocomposite. Figure 1a shows that the PPy-TiO_2_ nanocomposite contains C, O, N, and Ti elements. Figure 1b–e shows the maps of C, O, N, and Ti elements on the surface of PPy-TiO_2_ composites, respectively. As can be seen from the spectral diagram of Figure 1b C, there are three types of binding modes of element C. Peaks located at 284.8 eV, 286.47 eV, and 288.82 eV are considered to be characteristic peaks of the C=C&C-C bond, C-N bond, and C-O bond, respectively. The C=C&C-C and C-N bonds are mainly derived from pyrrole rings. The C-O bond mainly comes from the residual organic matter on the surface of TiO_2_. The peaks at 530.13 eV and 531.73 eV in Figure 1c O element spectrum are considered to be the characteristic peaks of O-Ti and O-H, respectively. The former corresponds to lattice oxygen in TiO_2_ and the latter to hydroxyl oxygen adsorbed on the surface of TiO_2_. The peaks at 399.97 eV and 402.09 eV in Figure 1d spectra of N elements correspond to the N-H bonds of neutral polypyrrole and positively charged nitrogen atoms. The presence of -N^+^- indicates that PPy components are in a doped state, and a chemical bond is established between PPy and TiO_2_, which improves the chemical stability of the composite [23]. Figure 1e shows the XPS spectrum of Ti2p, and the binding energy of Ti2p_3/2_ and Ti2p_1/2_ is 458.87 eV and 464.87 eV, respectively. It can be shown that the Ti element present on PPy-TiO_2_ is +4 valence, indicating that TiO_2_ is not reduced during the preparation of hydrogel, which is consistent with the results shown using XRD.

**Figure 2:**
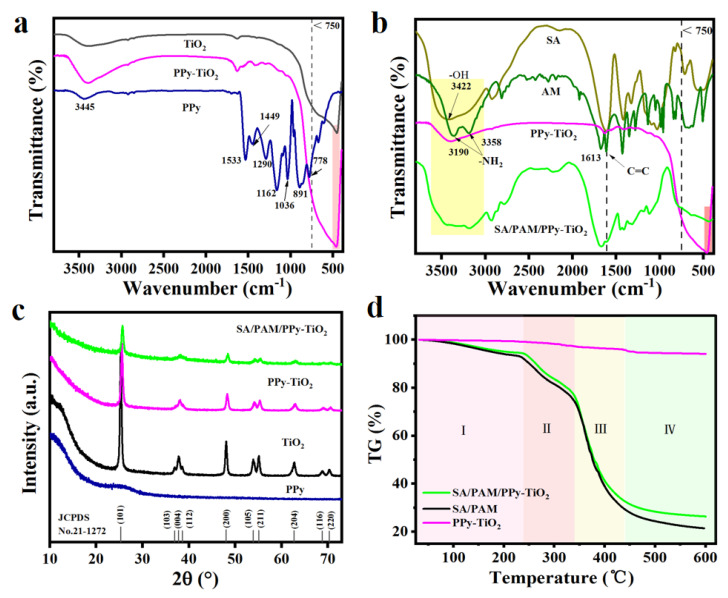
(**a**) FTIR spectra of TiO_2_, PPy-TiO_2_, and PPy; (**b**) FTIR spectra of SA, AM, PAM, PPy-TiO_2_, and SA/PAM/PPy-TiO_2_; (**c**) XRD spectra of PPy, TiO_2_, PPy-TiO_2,_ and SA/PAM/PPy-TiO_2_; (**d**) TG analysis graph of SA/PAM, and SA/PAM/PPy-TiO_2_.

Figure 2a shows the Fourier infrared spectroscopy (FTIR) of TiO_2_, PPy-TiO_2_, and PPy. It can be seen from the figure that the absorption peak near 3445 cm^−1^ corresponds to the N-H bond expansion vibration on the pyrrole ring. In addition, 1533 cm^−1^ and 1449 cm^−1^ are the characteristic peaks of the pyrrole ring, corresponding to the stretching vibration of the C=C/C-C bond and the stretching vibration of the C-N bond of the pyrrole ring, respectively. The peak at 1290 cm^−1^ corresponds to the =C-N bond stretching vibration peak on the pyrrole ring. Also, 1162 cm^−1^ 1036 cm^−1^ are the stretching vibration peaks of the =C-H bond. The absorption peaks at 891 cm^−1^ and 778 cm^−1^ correspond to the out-of-plane bending vibration peaks of C-H bonds. These characteristic peaks appear in the infrared spectrum of PPy-TiO_2_, indicating that PPy is successfully composited onto the surface of TiO_2_, and because the PPy content in the PPy-TiO_2_ composite material is very small, the characteristic absorption peak of PPy is not obvious. Figure 2b shows the infrared spectra of SA, AM, PAM, PPy-TiO_2,_ and SA/PAM/PPy-TiO_2_. The yellow region shows that the O-H, AM, and N-H dual-characteristic absorption peaks of PAM on SA are all reflected on SA/PAM/PPy-TiO_2_, but the peak shape is wider. This is what causes hydrogen bonds to form between the components. In the FTIR spectra of SA, the absorption peaks at 1029 and 1612 cm^−1^ are the absorption peaks of the C-O and -COO functional groups of SA, respectively, and the C-O functional groups are obviously reflected in the FTIR spectra of SA/PAM/PPy-TiO_2_. In the FTIR spectra of SA/PAM/PPy-TiO_2_, the symmetric stretching vibration absorption peaks of -COO- functional groups all move to the left to a larger wave number, indicating that ionic binding between Ca^2+^ and -COO^−^ exists in the composite hydrogel [37]. The C=C double bond at 1613 cm^−1^ in AM disappeared in the FTIR spectra of PAM and SA/PAM/PPy-TiO_2_, indicating that TiO_2_ successfully promoted the polymerization of AM monomers under solar radiation. In Figure 2a,b, in the red region of <750 cm^−1^ wave number range, both PPy-TiO_2_ and SA/PAM/PPy-TiO_2_ show strong TiO_2_ characteristic peaks, indicating that TiO_2_ has been successfully integrated into the composite material. This is also the reason why SA/PAM/PPy-TiO_2_ composite hydrogels have photocatalytic ability.

Figure 2c shows the X-ray powder diffraction (XRD) spectra of PPy, TiO_2_, PPy-TiO_2_, and SA/PAM/PPy-TiO_2_. The XRD pattern of PPy has a relatively wide diffraction peak near 2θ = 23°, which indicates that PPy is an amorphous substance. The XRD pattern of TiO_2_ reveals that the diffraction peaks at 2θ = 25.2°,36.9°, 37.8°, 38.5°, 48.0°, 53.8°, 55.0°, 62.6°, 68.7, and 70.3°were observed corresponding to the crystal planes (101), (103), (004), (112), (200), (105), (211), (204), (116), and (220) of the anatase phase TiO_2_ (JCPDS No. 21-1272), indicating that the crystalline structure of TiO_2_ remains unchanged during the formation process in both PPy-TiO_2_ and SA/PAM/PPy-TiO_2_ hydrogels, but the intensity of the peaks is reduced, which is due to the reduction in the TiO_2_ content. In addition, the one-to-one correspondence of good diffraction peaks indicates that TiO_2_ has been successfully integrated into the composite hydrogel and has no effect on the crystal shape of TiO_2_ in the process of preparation and drying, which also reveals the reason why SA/PAM/PPy-TiO_2_ composite hydrogel still has good photocatalytic performance.

The change in ambient temperature in different application scenarios is different, so it is necessary to explore the thermal stability of SA/PAM, PPy-TiO_2,_ and SA/PAM/PPy-TiO_2_ nanocomposite hydrogels at different temperatures. Figure 2d shows the thermogravimetric (TG) analysis curves of SA/PAM and SA/PAM/PPy-TiO_2_ composite hydrogels. It can be seen that the whole thermal decomposition process can be roughly divided into four stages. The first weight-loss stage of the composite hydrogel is at 25~237 °C, and the decrease in its weight is mainly due to the evaporation of water molecules in the composite hydrogel. Next, 25~337 °C is the second weight-loss stage of the composite hydrogel, and the weight loss is mainly due to the thermal degradation of functional groups such as hydroxyl, carboxyl, and amide in the composite hydrogel and the fracture of C-N bond in the MBA part of the hydrogel network. The weight loss in the third and fourth stages in the range of 337~437 °C and 437~600 °C is attributed to the breaking of polymer macromolecular chains at higher temperatures. When the temperature rises to 600 °C, the final residual ash mass of SA/PAM and SA/PAM/PPy-TiO_2_ composite hydrogels is 21.41 and 26.35% of the original mass, respectively. The thermogravimetric curves of SA/PAM and SA/PAM/PPy-TiO_2_ composite hydrogels show that the structure of the composite hydrogels is relatively stable at 237 °C, and the addition of PPy-TiO_2_ improves the thermal stability of the hydrogel matrix. The TG diagram of PPy-TiO_2_ reveals excellent thermal stability, with a weight loss of approximately 4.5% primarily attributed to the thermal decomposition of PPy.

### 3.2. Analysis of Solid Ultraviolet–Visible Diffuse Reflection Spectra

**Figure 3:**
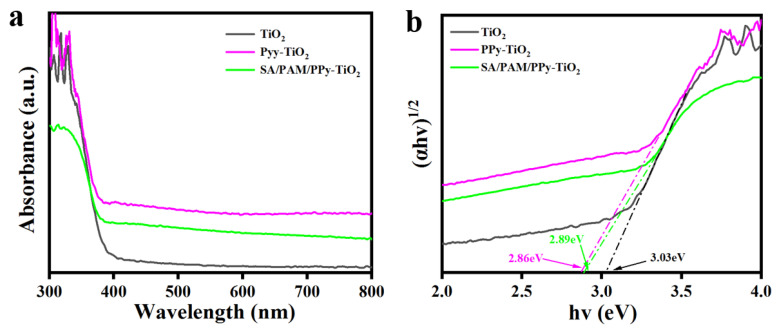
(**a**) UV-vis DRS of TiO_2_, PPy-TiO_2,_ and SA/PAM/PPy-TiO_2_; (**b**) forbidden band width maps of TiO_2_, PPy-TiO_2,_ and SA/PAM/PPy-TiO_2_.

In order to judge the light absorption capacity of the material, Figure 3a shows the solid ultraviolet–visible diffuse reflection spectra (UV-vis DRS) of TiO_2_, PPy-TiO_2_, and SA/PAM/PPy-TiO_2_ composite hydrogels. Pure TiO_2_ can only absorb ultraviolet light and has little response to visible light. Compared with pure TiO_2_, the absorption of visible light of the composite modified by PPy is greatly improved because the band gap of polypyrrole is narrow, and its spectral absorption range covers the entire UV-visible region. The bandgaps of TiO_2_, PPy-TiO_2,_ and SA/PAM/PPy-TiO_2_ composites were calculated using UV-VIS diffuse reflection spectra combined with Kubelka–Munk equation [38,39] and Eg = 1240/λ (Figure 3b), where α, h, and ν are the adsorption coefficient, Planck constant, and optical frequency, respectively. The band gaps of TiO_2_, PPy-TiO_2,_ and SA/PAM/PPy-TiO_2_ are 3.03, 2.86, and 2.89 eV, respectively. The presence of PPy reduces the band gap of TiO_2_, so the band gap energy value of the composite becomes smaller than TiO_2_, and it also has a strong absorption of visible light. Ultraviolet light only accounts for about 5% of solar energy, and how to effectively use the visible light in the sun is the focus of current research. PPy expands the spectral absorption range of TiO_2_ and makes the composite material have a visible light response, which is also the reason why SA/PAM/PPy-TiO_2_ composite hydrogels have excellent photocatalytic performance in sunlight.

### 3.3. Fluorescence Spectrum Analysis

**Figure 4:**
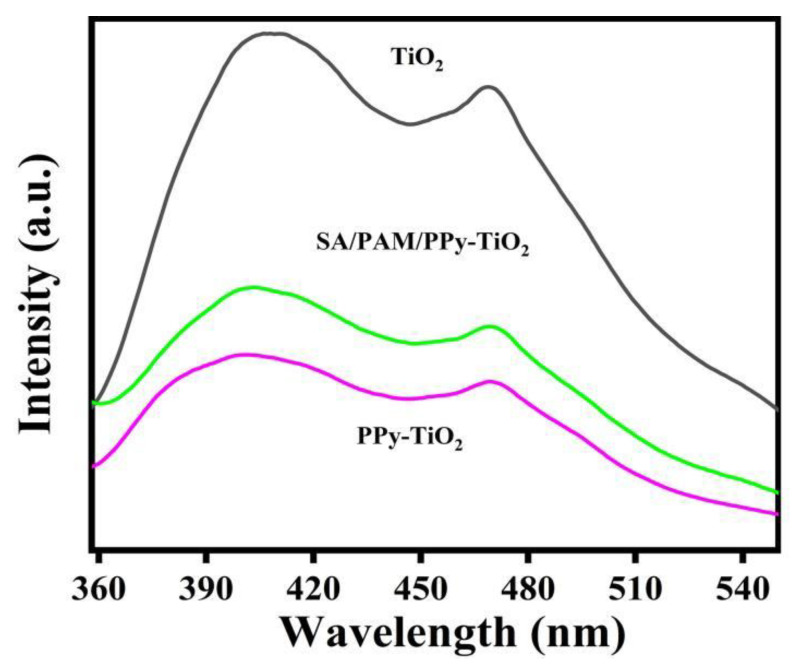
PL spectra of TiO_2_, PPy-TiO_2,_ and SA/PAM/PPy-TiO_2_.

Steady-state fluorescence spectroscopy was used to determine the recombination efficiency of photogenerated electron-hole pairs before and after modification. It is an important tool to evaluate the photocatalytic performance of photocatalysts. As shown in Figure 4, the emission frequency band of the fluorescence spectrum is 360~540 nm. A large number of studies have shown that after fluorescence generation, if the fluorescence spectral peak of the material is weak, the photogenerated electron-hole pair recombination probability of the material is low. Obviously, compared with pure TiO_2_, the fluorescence spectral peaks of the composite hydrogels of PPy-TiO_2_ and SA/PAM/PPy-TiO_2_ after modified PPy are lower, indicating that the photogenerated electron-hole pair recombination rate is lower under sunlight irradiation. This is because the surface sensitization of polypyrrole on TiO_2_ affects the transfer efficiency of photogenerated electrons, so SA/PAM/PPy-TiO_2_ shows good photocatalytic activity.

### 3.4. Analysis of the Microstructure of Materials

**Figure 5:**
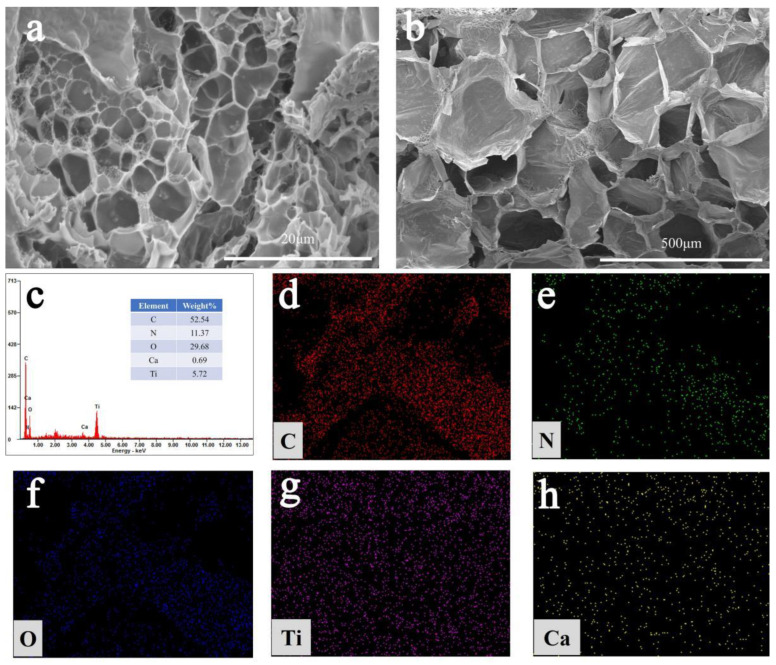
(**a**,**b**) are SEM images of SA/PAM/PPy-TiO_2_, (**c**) and (**d**–**h**) are EDS and mapping of SA/PAM/PPy-TiO_2_, respectively.

In order to study the microstructure of SA/PAM/PPy-TiO_2_ composite hydrogel, the composite hydrogel prepared by the experiment was fully swollen and freeze-dried, and then observed using field emission scanning electron microscopy (SEM), as shown in Figure 5. From Figure 5a,b, it can be seen that there are abundant micro-fold structures and hollow porous structures inside the composite hydrogel. These rich fold structures and pores provide a large number of adsorption sites, which are conducive to rapid adsorption of dyes, increase the probability of contact between dyes and photocatalytic active substances, and promote the photodegradation reaction of SA/PAM/PPy-TiO_2_ on dyes. EDS (Figure 5c) and mapping (Figure 5d–h) showed that the hydrogel contained five elements, C (52.54%), N (11.37%), O (29.68%), Ti (5.72%) and Ca (0.69%), and these five elements were evenly distributed in the SA/PAM/PPy-TiO_2_ composite hydrogel. The existence of the Ti element indicates the successful preparation of the SA/PAM/PPy-TiO_2_ composite hydrogel. In addition, the good dispersion of TiO_2_ particles in the composite hydrogel also makes the SA/PAM/PPy-TiO_2_ composite hydrogel exhibit more excellent photocatalytic performance.

**Figure 6:**
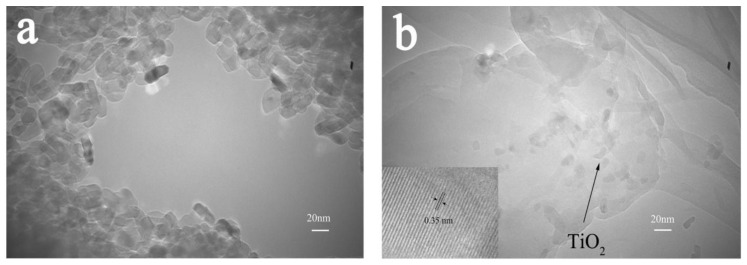
TEM spectra of (**a**) TiO_2_ and (**b**) SA/PAM/PPy-TiO_2_.

Figure 6 shows the high-power transmission electron microscopy (TEM) images of the TiO_2_ and SA/PAM/PPy-TiO_2_ composite hydrogel. It can be seen from the image that the diameter of TiO_2_ nanoparticles is about 25 nm, and the nanoparticles are agglomerated together with poor dispersion. The TEM images of SA/PAM/PPy-TiO_2_ in Figure 6b show the 3D fold structure of the composite hydrogel and TiO_2_ nanoparticles uniformly dispersed inside the composite hydrogel, indicating that the size of TiO_2_ nanoparticles did not change. The illustration of Figure 6b (shot after local magnification of the image) shows crystalline TiO_2_ nanoparticles with a lattice spacing of 0.35 nm, similar to anatase (101) planes [40], confirming that the TiO_2_ nanoparticles are embedded in the composite material, which is also the reason for the good photocatalytic performance of SA/PAM/PPy-TiO_2_.

### 3.5. Swelling Performance Analysis of Composite Hydrogel

**Figure 7:**
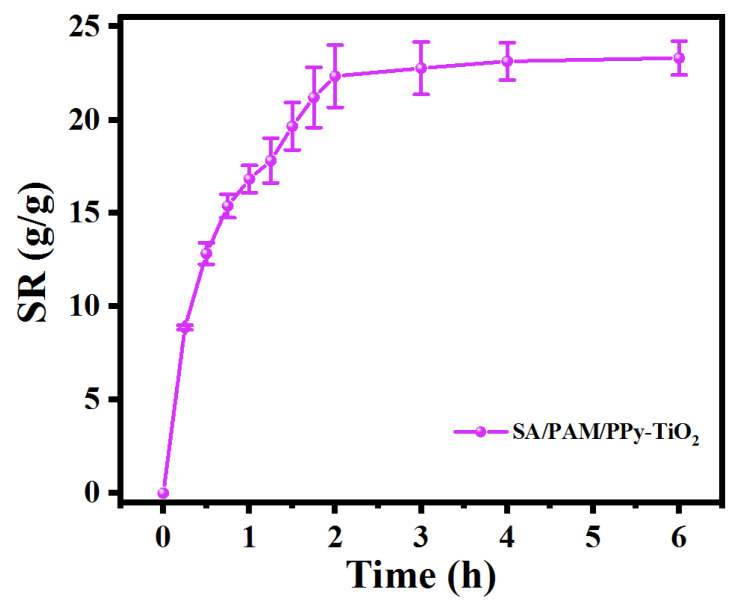
SA/PAM/PPy-TiO_2_ swelling rate graph.

The swelling curve of SA/PAM/PPy-TiO_2_ composite hydrogel in still water is shown in Figure 7. The swelling rate of the composite hydrogel increases rapidly at first, and a large number of water molecules enter the three-dimensional network structure of the hydrogel through diffusion so that the macromolecular chains of the hydrogel are fully extended. The volume of the hydrogel also increases with the increase in the swelling rate, and the adsorption equilibrium is reached at 2 h. Its value is 23.31 g/g. Good water absorption and swelling ensured that the composite hydrogel had excellent adsorption capacity. After swelling, the volume of the hydrogel changed to allow more solution to enter the hydrogel, which increased the probability of contact between organic dye molecules and the photoactive components on the surface of the hydrogel fiber, thus promoting the photocatalytic reaction.

### 3.6. Analysis of Photocatalytic Properties of Composite Hydrogels towards Methyl Orange and Methylene Blue

**Figure 8:**
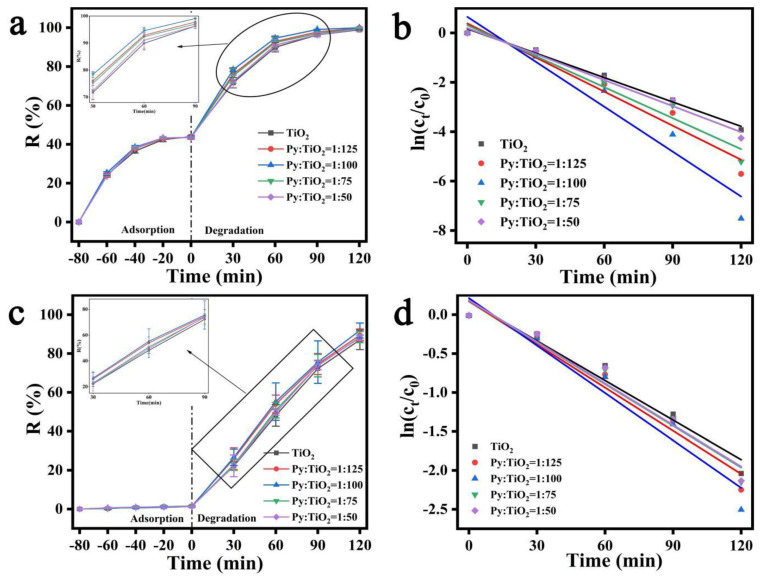
(**a**) Adsorption–photodegradation rate plots; (**b**) ln(c_t_/c_0_)~t relationship lines for MB by SA/PAM/PPy-TiO_2_ with different Py contents; (**c**) adsorption–photodegradation rate plots; (**d**) ln(c_t_/c_0_)~t relationship lines for MO by SA/PAM/PPy-TiO_2_ with different Py contents.

Figure 8 shows the different SA/PAM/PPy-TiO_2_ composite hydrogels prepared by changing the dosage ratio of Py to TiO_2_, so as to investigate the influence of different dosages of Py on the photocatalytic degradation of dyes MB and MO by SA/PAM/PPy-TiO_2_ composite hydrogels under sunlight. As can be seen from Figure 8a,c, both TiO_2_ containing pure TiO_2_ and TiO_2_ containing modified Py have good degradation effects on MB and MO under solar irradiation, but the photocatalytic activity of TiO_2_ composite containing modified Py is superior to that of pure TiO_2_, with an increase in the addition of Py. The photocatalytic activity of the composite particles increases first and then decreases. When the amount of Py and TiO_2_ is 1:100, the photocatalytic activity of the composite under sunlight is higher. This is because when the amount of polypyrrole is small, the polypyrrole on the surface of TiO_2_ is relatively small, the visible light in the solar light cannot be fully utilized, and the electrons transferred by polypyrrole to TiO_2_ are relatively small, so its photocatalytic activity under sunlight is poor. However, when the amount of polypyrrole is too much, the TiO_2_ surface will also be covered with too much polypyrrole, and electrons cannot be effectively transferred between polypyrrole and TiO_2_, which hinders the separation of photogenerated electrons and hole pairs, resulting in the decline of photocatalytic activity. Therefore, when the amount of Py and TiO_2_ substance is determined to be 1:100, SA/PAM/PPy-TiO_2_ composite hydrogel has the best photocatalytic efficiency under sunlight.

In the process of solar photocatalytic reaction, the evaluation of the photodegradation rate is also significant. The photodegradation process of MO and MB by SA/PAM/PPy-TiO_2_ composite hydrogel also conforms to the quasi-first-order kinetic process, and the formula [41] is as follows:

(3) $$\ln\left(c_{t}∕c_{0}\right)=-Kt$$

where c_0_ is the concentration of the pollutant at adsorption equilibrium, c_t_ is the concentration of the pollutant at time t, K is the quasi-first-order rate constant (min^−1^), and t is time (min). The K value is calculated separately from the slope of the graph of ln(c_t_/c_0_) and t for each photocatalyst. Table 1 and Table 2 list the quasi-first-order rate constant K and the correlation coefficient R^2^ of the photodegradation of MB and MO, respectively under different conditions, and both R^2^ are greater than 0.94. The fitting curves of ln(c_t_/c_0_) and t (Figure 8b,d) also have a good linear relationship. The results indicate that the photodegradation of MO and MB by SA/PAM/PPy-TiO_2_ composite hydrogel was consistent with the quasi-first-order reaction kinetics equation. When the amount of PPy and TiO_2_ was 1:100, the maximum degradation rate constant K was obtained, which further indicates that the photocatalytic performance of composite hydrogels was the best after the addition of PPy at this ratio.

**Figure 9:**
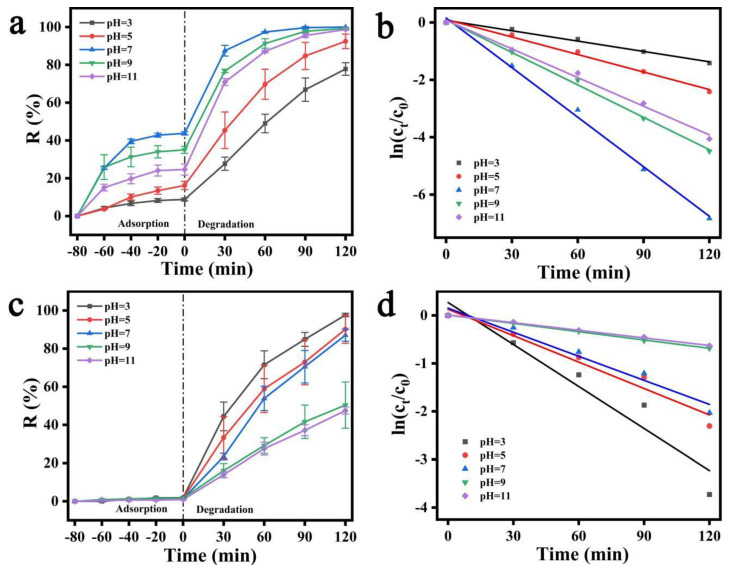
(**a**) Adsorption–photodegradation rate plot; (**b**) ln(c_t_/c_0_)~t relationship line of SA/PAM/PPy-TiO_2_ on MB in different pH environments; (**c**) adsorption–photodegradation rate plot; (**d**) ln(c_t_/c_0_)~t relationship line of SA/PAM/PPy-TiO_2_ on MO in different pH environments.

The pH value of dye wastewater in the natural environment is also constantly changing, so it is also important to study the effect of pH on the photocatalytic degradation activity of dye MB and MO under sunlight. Figure 9 illustrates the effects of different pH environments on the degradation of dyes MO and MB by SA/PAM/PPy-TiO_2_ composite hydrogels. The pH value of the dye solution was adjusted by HCl and NaOH. For dye MB (Figure 9a), as the pH value increased, the adsorption efficiency of SA/PAM/PPy-TiO_2_ composite hydrogel on the dye MB first increased and then decreased, and the adsorption efficiency was the highest when pH = 7, with a value of 43.73%. The high adsorption efficiency enables MB molecules to gather around PPy-TiO_2_, which increases the probability of hydroxyl radical contact with MB molecules, thus promoting the photocatalytic reaction. Under acidic conditions, a large amount of H^+^ in the solution system protonated the -COO^−^ and -NH_2_ functional groups on the surface of the composite hydrogel to form -COOH and -NH_3_^+^, which made the surface of the composite hydrogel positively charged, which hindered the adsorption of alkaline dye MB (-C=N^+^) and reduced the contact probability between MB molecules and the photocatalytic active components, so the photocatalytic efficiency was low. When pH > 7, a large number of Na^+^ in the solution will compete with dye molecules for adsorption sites inside the hydrogel, hindering the electrostatic attraction of -COO^−^ groups to MB molecules, thus reducing the adsorption of MB, and inhibiting the photocatalytic reaction.

For MO (Figure 9c), the photocatalytic activity of SA/PAM/PPy-TiO_2_ continuously decreased with an increase in pH value. This is because the molecular structure formula of MO is different in different pH environments. In acidic conditions, MO is a quinone structure, and in alkaline conditions, MO is an azo structure. Most of the literature also show that the degradation effect of quinone structure is much better than that of azo structure; that is, the degradation effect of MO in an acidic environment is better than that in an alkaline environment. In addition, the surface of the composite hydrogel is positively charged under acidic conditions, while MO has a quinone structure and the sulfate end of its molecule is negatively charged, so MO can be more easily adsorbed into the hydrogel to participate in the photocatalytic degradation reaction.

The degradation of dyes MO and MB by SA/PAM/PPy-TiO_2_ composite hydrogels in different pH environments also conforms to the quasi-first-order kinetic process. Figure 9b,d fitted the kinetic curves of each reaction and found that ln(c_t_/c_0_) of each reaction had a good linear relationship with t, and R^2^ were all greater than 0.92, indicating that the photodegradation process of MO and MB by SA/PAM/PPy-TiO_2_ composite hydrogel was consistent with the quasi-first-order reaction kinetic equation under different pH conditions. When pH = 7, the degradation rate constant K of the composite hydrogels for MB is the highest, which further indicates that the photocatalytic performance of the composite hydrogels is the best at this pH. When the degradation rate constant K of the composite hydrogel for MO in an acidic environment is greater than that in an alkaline environment, it is confirmed that acidic conditions are conducive to MO degradation. Table 3 shows a comparative study of various photocatalysts on the photodegradation performance of dyes, indicating that SA/PAM/PPy-TiO_2_ has a faster photocatalytic rate and is easy to recycle.

**Figure 10:**
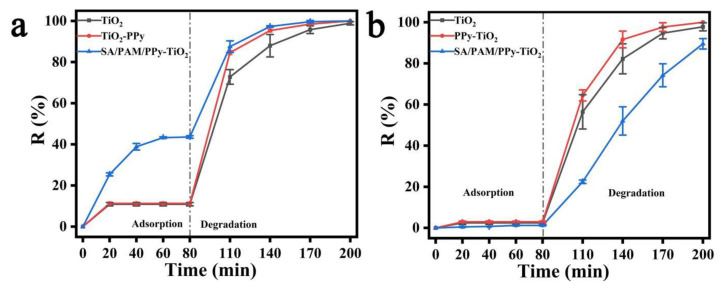
Adsorption–photodegradation rates of different dyes by TiO_2_, PPy-TiO_2,_ and SA/PAM/PPy-TiO_2_ graphs (**a**) MB; (**b**) MO.

Figure 10 shows the photocatalytic degradation rates of dyes MB and MO by nano-TiO_2_, PPy-TiO_2,_ and SA/PAM/PPy-TiO_2_ composite hydrogels under sunlight. It can be seen from Figure 10a,b that composite hydrogel has the best photocatalytic degradation activity for MB under sunlight, followed by PPy-TiO_2_, and pure TiO_2_ has the worst. For MO dye, the photocatalytic degradation activity of PPy-TiO_2_ is the best, followed by TiO_2_, and SA/PAM/PPy-TiO_2_ is the worst. This is because TiO_2_ modified by Py has a better absorption of visible light while inhibiting the photogenic carrier recombination, so the photocatalytic performance of PPy-TiO_2_ under sunlight is better than that of pure TiO_2_. SA/PAM/PPy-TiO_2_ has good adsorbability for MB, and it can quickly adsorb MB molecules in the solution and aggregate around the photocatalytic active ingredient PPy-TiO_2_, which is conducive to the rapid degradation of MB by the generated free radicals. On the contrary, SA/PAM/PPy-TiO_2_ has poor adsorption of MO, so only a small amount of MO molecules can be adsorbated to the inside of SA/PAM/PPy-TiO_2_ composite hydrogel, and the photocatalytic reaction is mostly carried out on its surface, so it has the worst photocatalytic degradation activity compared with TiO_2_ and PPy-TiO_2_. However, SA/PAM/PPy-TiO_2_ composite hydrogels have the advantages of easy recovery and separation compared with nano-photocatalytic materials, which is also an important indicator to evaluate the overall performance of photocatalysts.

### 3.7. Analysis of Photocatalytic Performance of Composite Hydrogels for Different Dyes

**Figure 11:**
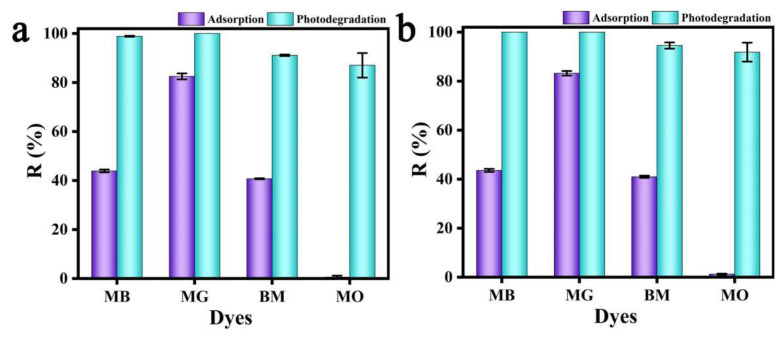
Adsorption and photodegradation efficiency of (**a**) SA/PAM/TiO_2_ and (**b**) SA/PAM/PPy-TiO_2_ for different dyes.

Photocatalytic oxidation technology is universal to the photodegradation of organic pollutants in aqueous solution and can photocatalyze the degradation of different organic pollutants. Therefore, photocatalytic oxidation technology has a broad prospect in the treatment of dye wastewater. In order to study the degradation effect of SA/PAM/TiO_2_ and SA/PAM/PPy-TiO_2_ composite hydrogels on other pollutants, the composite hydrogels were used to photocatalytically degrade MB, mg, BM, and MO dyes with a concentration of 10 mg/L, respectively. As can be seen from Figure 11, the degradation efficiency of the two kinds of composite hydrogels on alkaline dyes MB, MG, and BM is higher than that of acidic dyes MO. This is because alkaline dyes can be ionized into positively charged dye molecules during the reaction and generate electrostatic attraction with negatively charged carboxylic acid groups on the composite hydrogels, which increases the probability of their contact with photocatalytic active components. The photocatalytic reaction was further improved. MO is an acidic dye, which can be ionized into positively charged dye molecules in the reaction, which produces electrostatic repulsion with the negatively charged carboxylic acid group on the complex hydrogel, and the degradation rate is lower than the other three kinds. The degradation rates of MB, MG, BM, and MO by SA/PAM/TiO_2_ composite hydrogels were 98.89%, 100%, 91.13%, and 87.05%, respectively. The highest degradation rates of SA/PAM/PPy-TiO_2_ for MB and MG were 100%, and the degradation rates of BM and MO decreased successively, which were 95.33% and 91.85%, respectively. It can be seen that the degradation efficiency of SA/PAM/PPy-TiO_2_ for different dyes is higher than that of SA/PAM/TiO_2_ composite hydrogels. This is because the introduction of PPy increases the absorption of visible light in solar rays by the composite hydrogel, which in turn promotes the rate of photocatalytic reaction.

### 3.8. Analysis of Photocatalytic Performance of Composite Hydrogels for Mixed Dyes

**Figure 12:**
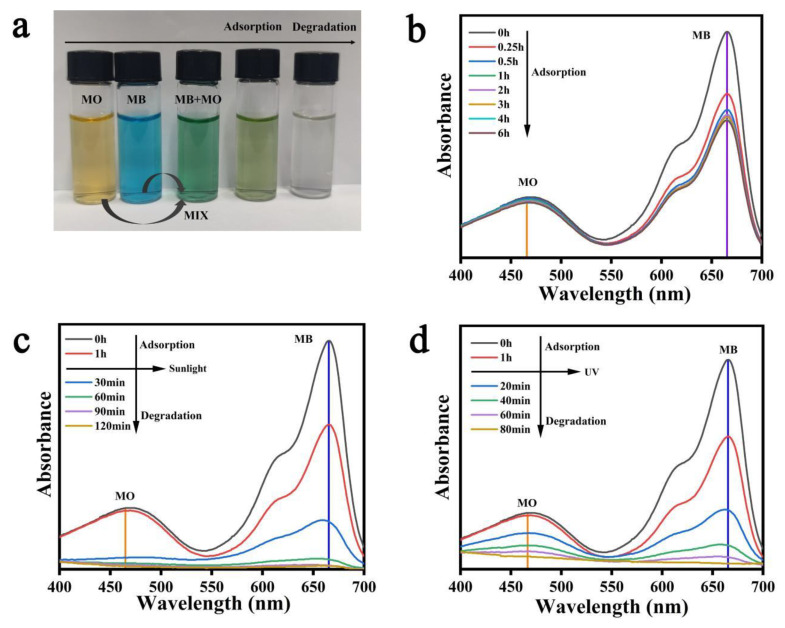
(**a**) Pictures of adsorption–photodegradation of mixed dyes by SA/PAM/PPy-TiO_2_; (**b**) UV-Vis spectra of MO + MB mixed dye solutions after the adsorption by SA/PAM/PPy-TiO_2_; UV-Vis spectra of MO + MB mixed dye solutions after the degradation by SA/PAM/PPy-TiO_2_ under (**c**) sunlight and (**d**) UV light.

Organic dye wastewater may contain a variety of harmful dyes, such as acidic dyes MO and alkaline dyes MB. The molecular structure of MB and MO is different because they have different reaction rates in the photocatalytic reaction process. Therefore, it is necessary to study the photocatalytic degradation behavior of different components in MB and MO mixed solution by SA/PAM/PPy-TiO_2_ composite hydrogels. Figure 12a shows that the color of the mixture of MO and MB is green, and the color of the solution becomes yellow-green after adsorption by the composite hydrogel, which is the reason for the decrease in the concentration of MB components in the mixture. The characteristic peak of dye MB (664 nm) in the UV-VIS spectrum decreased significantly (Figure 12b), which also indicated that the composite hydrogel had a good adsorption effect on MB. The characteristic peak of dye MO (465 nm) remained almost unchanged, indicating that the adsorption effect of composite hydrogel on MO was poor. Figure 12c,d show the UV-VIS spectra of composite hydrogel degradation of mixed dyes under sunlight and LED UV lamps, respectively. The degradation rate of MB and MO components in the mixed dyes was about the same under sunlight, but the degradation rate of MB was significantly faster than that of MO under ultraviolet irradiation. This difference may be caused by the fact that the penetration ability of solar rays to the composite hydrogel is less than that of ultraviolet light emitted by ultraviolet lamps. PPy-TiO_2_ attached to the surface of the composite hydrogel absorbs most of the sunlight, and most MO and a small part of MB in the solution participate in the surface photocatalytic reaction of the composite hydrogel, while the interior of the composite hydrogel receives less solar light. Therefore, MB molecules adsorbated in the hydrogel degrade slowly, so the degradation rate of MO and MB in the mixed dye under sunlight is roughly the same. However, the light emitted by the ultraviolet lamp has a strong penetration ability, and the PPy-TiO_2_ inside the hydrogel can fully absorb the energy of the light and then participate in the photodegradation reaction, speeding up the degradation rate of MB molecules adsorbing into the hydrogel. Therefore, the photodegradation efficiency of MB is faster than that of MO under the irradiation of the ultraviolet lamp.

### 3.9. Mechanical Properties and Macroscopic Morphology Analysis of Composite Hydrogels

**Figure 13:**
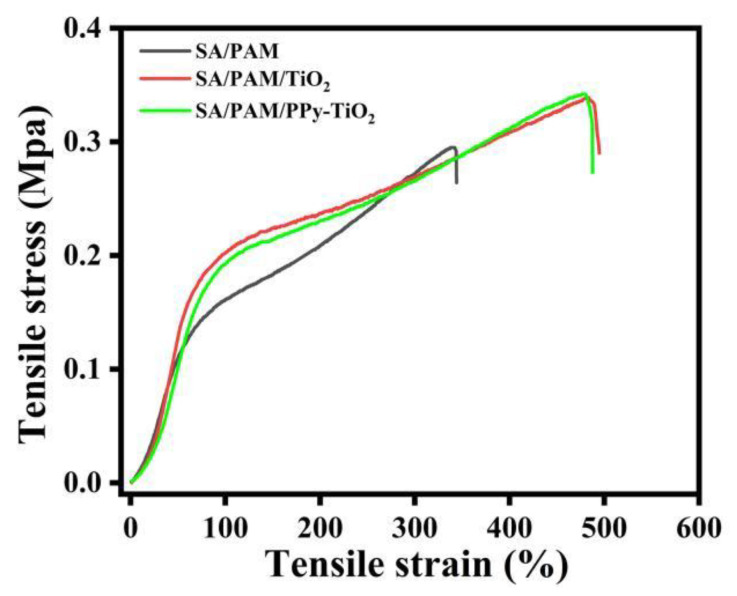
Tensile stress–strain curves of SA/PAM, SA/PAM/TiO_2,_ and SA/PAM/PPy-TiO_2_.

The mechanical properties of SA/PAM, SA/PAM/TiO_2,_ and SA/PAM/PPy-TiO_2_ composite hydrogels were tested by an electronic universal tensile testing machine, and the results are shown in Figure 13. The stress and strain of SA/PAM/PPy-TiO_2_ composite hydrogels are 0.342 Mpa and 479%, respectively. Compared with SA/PAM hydrogel (stress 0.295 Mpa, strain 340%), its mechanical properties improved significantly. This is because the presence of inorganic nanoparticles TiO_2_ can effectively limit the sliding and stretching of polymer chains, and enhance the strength of hydrogel matrix. Compared with SA/PAM/TiO_2_ composite hydrogel (Figure 13, stress 0.338 Mpa, strain 480%), the tensile stress–strain curve did not change significantly, indicating that the addition of trace polypyrrole did not change the internal structure of the composite hydrogel. SA/PAM/PPy-TiO_2_ composite hydrogel still has good mechanical properties.

**Figure 14:**
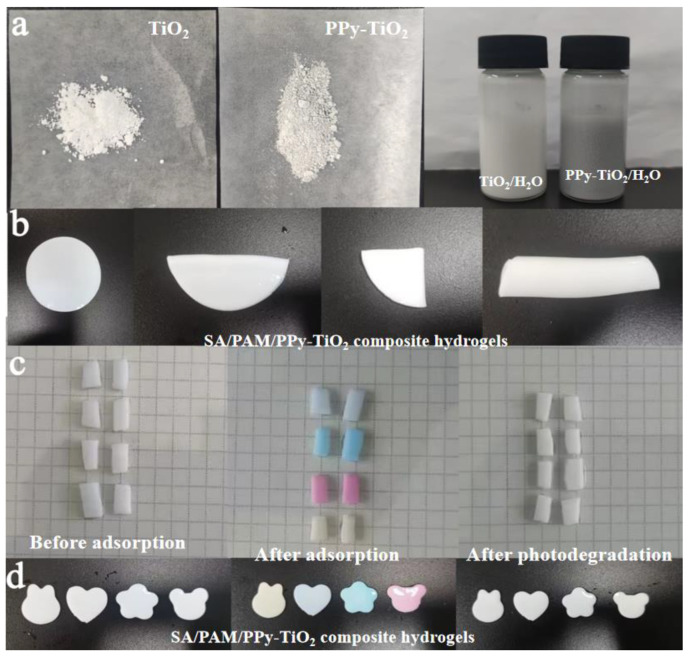
Macroscopic morphology of (**a**) TiO_2,_ PPy-TiO_2,_ TiO_2_/H_2_O, and PPy-TiO_2_/H_2_O; (**b**) different shapes of SA/PAM/PPy-TiO_2_ composite hydrogels; (**c**,**d**) different shapes before and after adsorption–photodegradation.

Figure 14 shows the macro morphology of the prepared PPy-TiO_2_ and SA/PAM/PPy-TiO_2_ composite hydrogels. It can be seen from Figure 14a that after PPy modification, TiO_2_ powders and water dispersions are both gray and white, indicating that Py was successfully polymerized on the surface of TiO_2_ particles. In Figure 14b, the SA/PAM/PPy-TiO_2_ prepared shows a white surface, which was due to the small content of PPy. However, it can be seen that TiO_2_ on the surface of the composite hydrogel is uniformly dispersed and has good stability because the surface of TiO_2_ particles contains a hydrophilic functional hydroxyl group, which can make nano TiO_2_ firmly fixed on the hydrogel fiber through hydrogen bonding, and folding, stretching and soaking the composite hydrogel will not make it fall off. Figure 14c,d show the surface color changes of the composite hydrogels cut into different shapes before and after adsorption–photodegradation. It can be seen that after adsorption, the surface of the hydrogels showed different colors of dyes, and these colors disappeared after photocatalytic degradation, while the composite hydrogels remained intact. This is due to the good stability of SA/PAM/PPy-TiO_2_ composite hydrogels.

### 3.10. Recycle Performance Analysis of Composite Hydrogels

**Figure 15:**
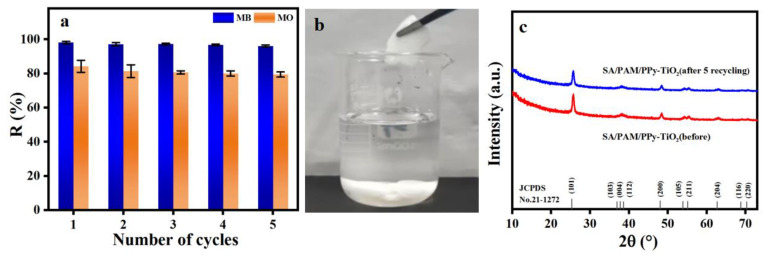
(**a**) The recycling performance of SA/PAM/PPy-TiO_2_ hydrogel; (**b**) the recovery process of SA/PAM/PPy-TiO_2_ hydrogel; (**c**) XRD spectra of SA/PAM/PPy-TiO_2_ hydrogel before and after 5 cycles.

The recycling performance of catalysts is an important index to evaluate the stability and practical application of photocatalysts. In order to evaluate the photochemical stability and recyclability of SA/PAM/PPy-TiO_2_ composite hydrogel, the same SA/PAM/PPy-TiO_2_ composite hydrogel was used to photodegrade MB and MO in five cycles. After each experiment, the composite hydrogel was simply soaked and cleaned with distilled water. As shown in Figure 15a, after five cycle tests, the degradation efficiency of SA/PAM/PPy-TiO_2_ composite hydrogel for MB was almost unchanged, and the degradation efficiency for MO was slightly decreased, but the degradation rate was still 79.38%. Figure 15b shows that the SA/PAM/PPy-TiO_2_ composite hydrogel can be separated from the solution very simply and can be used many times in practical applications. The XRD spectra of SA/PAM/PPy-TiO_2_ hydrogel were analyzed before and after 5 cycles to evaluate the stability of the photocatalysts, as depicted in Figure 15c. After 5 cycles, all the diffraction peaks of the SA/PAM/PPy-TiO_2_ hydrogel were similar to that before the cycle, but the intensity of the diffraction peak was slightly reduced due to the decrease of TiO_2_ content. This result proves that the photocatalyst has good stability.

### 3.11. Analysis of Photocatalytic Mechanism of Composite Hydrogels

**Figure 16:**
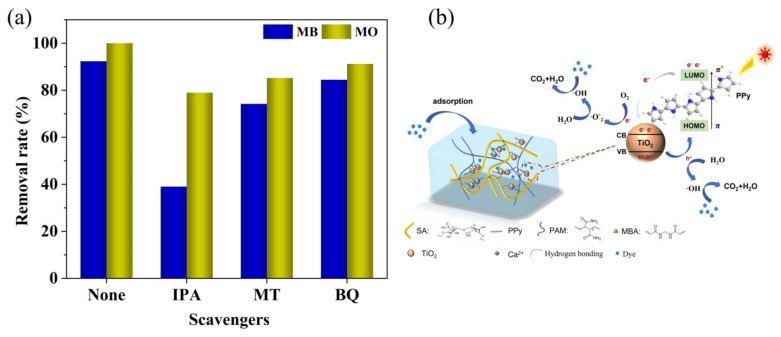
(**a**) The effect of different radical trapping agents on the efficiency of degrading dyes, and (**b**) a schematic diagram of the photodegradation mechanism of dyes by SA/PAM/PPy-TiO_2_.

In order to study the photocatalytic degradation of the main active species of MB/MO, we conducted a series of capture tests on SA/PAM/PPy-TiO_2_ under sunlight. As shown in Figure 16, isopropyl alcohol (IPA), potassium iodide (KI), and p-benzoquinone (BQ) were used as ·OH, h^+^, and ·O_2_^−^ scavengers, respectively. After adding BQ, the degradation rate was similar to that without adding a trapping agent, indicating that ·O_2_^−^ non-MB/MO photodegrades the main active species. However, IPA and KI have a great influence on the photocatalytic activity of the catalysts, indicating that ·OH and h^+^ are the main active species in the photocatalytic degradation of MB/MO. Based on the research results of scavengers, the possible mechanism of the SA/PAM/PPy-TiO_2_ composite hydrogel to enhance the catalytic activity of visible light is described by the following equation [18,19,38]:

(4) $$PPy-TiO_{2}+hv\to PPy\left(e_{LUMO}^{-}+h_{HOMO}^{+}\right)-TiO_{2}$$

(5) $$H_{2}O+h^{+}\to \cdot OH$$

(6) $$O_{2}+e^{-}\to \cdot O_{2}^{-}$$

(7) $$\cdot O_{2}^{-}+H_{2}O\to \cdot OH$$

(8) $$\cdot OH+MO/MB\to CO_{2}+H_{2}O$$

(9) $$\cdot O_{2}^{-}+MO/MB\to CO_{2}+H_{2}O$$

The photocatalytic process typically involves three main steps: absorption of light by the photocatalyst, promotion of valence band (VB) electrons to the conduction band (CB) across the band gap, resulting in the separation of photogenerated carriers such as electron-hole pairs (e^−^/h^+^) at the surface of photocatalyst, and facilitation of an interfacial photocatalytic redox reaction [45,46]. When UV/visible light illuminates PPy, photons with energies higher than the band gap excite electrons from the VB to the CB. This phenomenon is called π-π* electron transition. Compared to TiO_2_, PPy has a lower band gap, so PPy acts as a photosensitizer to absorb a wide range of visible light. The e^-^ in the lowest unoccupied molecular orbital (LUMO) of the PPy chain is injected with CB of TiO_2_, which reacts with the adsorbed O_2_ molecule to form ·O_2_^−^, while the hole may form ·OH with water. ·O_2_^−^ radicals can also form ·OH radicals by reacting with H_2_O. ·OH radical is the main oxidizing substance in the photodegradation process and has strong activity. ·OH and ·O_2_^−^ can promote MB/MO to be oxidized and decomposed to form small molecules such as CO_2_ and H_2_O.

## 4. Conclusions

The SA/PAM/PPy-TiO_2_ nanocomposite hydrogel photocatalyst with visible light response was synthesized via in situ polymerization. XPS analysis revealed the formation of chemical bonds between PPy and TiO_2_, indicating successful polymerization of PPy on the surface of TiO_2_ particles. UV-vis DRS results demonstrated that the composite hydrogel exhibited excellent absorption capacity for visible light, extending its light absorption range to the visible region upon incorporation of TiO_2_, thereby significantly enhancing sunlight utilization efficiency. The photocatalytic performance of SA/PAM/PPy-TiO_2_ composite hydrogels towards MO and MB degradation under sunlight irradiation was investigated at different PPy to TiO_2_ dosage ratios. Notably, when the PPy to TiO_2_ ratio was 1:100, the SA/PAM/PPy-TiO_2_ nanocomposite hydrogels showed superior photocatalytic efficiency. After 120 min of sunlight exposure, the degradation efficiency reached approximately 100% for MB and 91.85% for MO. Compared to nano-TiO_2_ and PPy-TiO_2_ counterparts, the composite hydrogel exhibited a higher degradation rate for MB dye under sunlight irradiation while maintaining ease of separation and recovery. Furthermore, even after five cycles, there was no significant decrease in the photocatalytic efficiency observed for the composite hydrogel. In conclusion, this study presents a novel approach for designing and synthesizing adsorption–photocatalytic functional composite hydrogels with high-performance characteristics such as stability and visible light responsiveness. These findings hold great practical significance in addressing current environmental pollution challenges by providing insights into constructing highly efficient and stable photocatalytic materials.

## Figures and Tables

**Table 1:** Primary reaction kinetic parameters of SA/PAM/PPy-TiO_2_ composites for the photodegradation of MB under different conditions.

Factor		K	R^2^
Py:TiO_2_	0	0.03292	0.99165
	1:125	0.04598	0.9569
	1:100	0.06062	0.93871
	1:75	0.04181	0.95925
	1:50	0.03509	0.98427
pH	3	0.01203	0.98872
	5	0.02036	0.99164
	7	0.05759	0.99693
	9	0.03767	0.99685
	11	0.03331	0.99392

**Table 2:** Primary reaction kinetic parameters of SA/PAM/PPy-TiO_2_ composites for the photodegradation of MO under different conditions.

Factor		K	R^2^
Py:TiO_2_	0	0.01696	0.95721
	1:125	0.0185	0.96055
	1:100	0.02032	0.94372
	1:75	0.01787	0.95833
	1:50	0.01782	0.95735
pH	3	0.02916	0.9256
	5	0.01837	0.95797
	7	0.01672	0.96463
	9	0.00577	0.99896
	11	0.00529	0.99841

**Table 3:** The degradation ability of SA/PAM/PPy-TiO_2_ was compared with other photocatalysts reported in the literature.

No.	Materials	Irradiation Time—Illumination Source	MO/MB Degradation Efficiency	References
1	PPy-TiO_2_	90 min—Sunlight	93% MB	[42]
2	PPy-TiO_2_	180 min—Sunlight	80% MO	[43]
3	PPy-TiO_2_	120 min—Sunlight	83% MO	[44]
4	SA/PAM/PPy-TiO_2_	120 min—Sunlight	100% MB/91.85% MO	Present study

## Data Availability

Data will be made available upon request.
